# Coordinating Fertility Preservation in Children and Young Adults at Risk of Treatment‐Induced Infertility: A Commentary

**DOI:** 10.1111/1471-0528.70238

**Published:** 2026-04-22

**Authors:** Ursula Blyth, Pedro Melo, Rod T. Mitchell, Suzannah A. Williams, Ephia Yasmin, Phillipa Sangster, Kokila Lakhoo, Sheila Lane, Richard A. Anderson, Christian M. Becker

**Affiliations:** ^1^ Nuffield Department of Women's and Reproductive Health University of Oxford Oxford UK; ^2^ Centre for Reproductive Health, Institute for Regeneration and Repair University of Edinburgh Edinburgh UK; ^3^ Department of Women's Health University College London Hospitals London UK; ^4^ Reproductive Medicine Unit, Elizabeth Garrett Anderson Wing University College London Hospitals NHS Trust London UK; ^5^ Nuffield Department of Surgical Sciences, Medical Sciences Division University of Oxford Oxford UK

**Keywords:** assisted conception, fertility preservation, infertility

Every year globally, about 400 000 new cancer cases are diagnosed in children and adolescents [1]. Survival rates for childhood cancer have improved dramatically since the 1970s, with 10‐year survival now estimated at 80% [2]. Children and young adults (CYA) (individuals under 25 years old) diagnosed with cancer or certain medical or genetic conditions are at high risk of infertility. Without proactive measures, individuals may lose their chance to have biological children. Survivors report infertility as one of the most distressing health consequences of their treatment, contributing to feelings of isolation and low self‐worth [3]. Yet fertility preservation treatment is not being offered to all eligible patients [4].

Recent years have seen increased advocacy and recognition of the importance of oncofertility, with national and international guidelines unanimously recommending that patients, parents and carers are informed about fertility preservation options as early as possible [4, 5]. We highlight the urgent need for nationally commissioned fertility preservation services and call on clinicians to recognise and prioritise the potential implications of medical treatments on future reproductive potential of children and young people [6].

Chemotherapy, especially alkylating agents, abdominopelvic radiotherapy, and certain newer agents such as immune checkpoint inhibitors, used to treat malignant and non‐malignant conditions (e.g., prior to haematopoietic stem cell transplant), confer a high risk of infertility [7, 8]. For post‐pubertal people, freezing sperm, eggs or embryos is the preferred method of fertility preservation. However, these options are not available for prepubertal children or adolescents who lack mature gametes, capacity to consent for gamete storage, or for whom the invasive nature of egg retrieval is not appropriate. Similarly, some older patients, in whom the need for urgent treatment does not allow time for ovarian stimulation, may be excluded [6]. In such cases, ovarian or testicular tissue cryopreservation constitutes the only fertility preservation option, provided the service is accessible and has the capacity to act at short notice [7]. Without access to gonadal tissue cryopreservation services, future fertility and the chance to have genetically related children, is at risk of being irretrievably lost once gonadotoxic treatment has been administered. This loss compounds the psychological burden of receiving a serious diagnosis and deprives young people and their families of hope and choice at a time when they are already navigating immense uncertainty.

Ovarian tissue cryopreservation (OTC) involves surgically retrieving part of or an entire ovary. The ovarian cortex, containing the primordial follicles, is then cryopreserved. When the individual wishes to have biological children, ovarian tissue is re‐transplanted into the remaining ovary or adjacent peritoneal pockets to restore ovarian endocrine and reproductive function, potentially enabling natural conception or medically assisted reproduction. By restoring endocrine function, OTC can also reduce the associated costs and potential health risks of long‐term hormone replacement therapy for women who develop premature ovarian insufficiency. In 2021, six major international centres reported that since 2004 over 200 children have been born following OTC [9]. Success rates of ovarian tissue re‐transplantation are approximately 90% for restoration of endocrine function and 30%–35% for live births [9]. International fertility preservation guidelines recognise OTC as an established fertility preservation technique [4].

Testicular tissue cryopreservation involves an open testicular biopsy to obtain tissue for freezing. Proof‐of‐principle that autotransplantation of cryopreserved prepubertal testicular tissue can generate sperm and result in live birth has been shown in primates; however, it is yet to be proven in humans [10]. This is likely to change soon: the first human re‐transplantation of testicular tissue has been reported [11], and a UK clinical research collaboration has received approval for a clinical trial [12]. Currently, testicular tissue is stored in the hope that future scientific progress will allow for the use of testicular tissue to generate sperm for use in medically assisted reproduction.

Ovarian and testicular tissue cryopreservation are specialised processes requiring expert teams and dedicated resources. Despite the existence of these services in many countries, significant gaps remain in coordination, funding and long‐term oversight. Health disparities such as age, ethnicity, socioeconomic status and geographical location can affect access to fertility services [13]. Ensuring equitable access requires national coordination with sustainable funding, clear referral pathways and governance frameworks.

Funding of fertility preservation depends on a country's healthcare model. For insurance based systems, such as the United States, coverage for fertility preservation for medical reasons is inconsistent and costs may fall on individuals and their families. This creates financial barriers that exacerbate socioeconomic inequalities. Insurance mandates in some states have improved access but significant geographic and socioeconomic variation remains [14]. Germany introduced reforms in 2019 requiring insurance coverage for medically indicated fertility preservation in an attempt to improve equity of access [15]. Long term funding of storage can also be contentious, particularly in children where tissue may be stored for decades. In publicly funded healthcare systems such as in Canada and the United Kingdom, competing healthcare priorities and the absence of nationally commissioned services can result in discretionary local funding or reliance on charitable support.

In the United Kingdom a hub and spoke model has been in place to reduce geographical variation in access to services. Specialist centres coordinate with patients' local hospitals to store ovarian or testicular tissue while patients continue to receive local care, close to home. This structure allows large portions of the UK to benefit from access to highly specialised centres with the facilities and expertise required for successful gonadal tissue cryopreservation. These services have enabled tissue storage for over 2700 CYAs in the UK and amongst European centres 95 live births have been reported following ovarian tissue autotransplantation [9, 16], demonstrating the potential of nationally coordinated services.

Fertility preservation services also require clear legal frameworks to navigate ethical issues that arise with storage of reproductive tissue including: consenting children, long term storage responsibilities, disposal procedures and management of stored tissue after the death of an individual. In France, fertility preservation is written into the public health code: healthcare providers must inform patients of fertility implications of treatment and refer for fertility preservation where appropriate. Despite this, referral is inconsistent, suggesting that legislation alone is not sufficient [17]. Education of healthcare professionals is key to ensure early counselling and referral of eligible patients. A coordinated, national approach to oncofertility care would ensure standardised pathways and transitions of care for all patients.

Oncofertility networks have been shown to increase pregnancy rates in cancer survivors [18]. Globally, four countries have integrated oncofertility networks into public health policy: the United States of America, Canada, Brazil and Australia [18]. The FertiPROTEKT network coordinates fertility preservation services in German‐speaking European countries [18], and NORDFERTIL offers a similar service across the Nordic region [19]. Only a small number countries maintain fertility preservation registries: Japan, Australia, and the FertiPROTEKT network. Prospective registries to capture uptake, complications and long term outcomes will enable monitoring for quality control as well as research. International collaboration allows sharing of clinical expertise and innovations. Established networks can support emerging services in low and middle income countries with training and protocol development.

Attention must also be given to ensuring that fertility preservation services are able to provide long‐term care. Survivorship following cancer requires multidisciplinary follow‐up to ensure long‐term physical, psychosocial, and reproductive health. Young girls and women require age‐appropriate, specialist gynaecology care to identify and manage late‐effects of treatments on reproductive health. This includes monitoring ovarian reserve, counselling about premature ovarian insufficiency, and ensuring appropriate hormone replacement therapy treatments if required. Similarly, young men require endocrine support and education about future fertility options after gonadotoxic treatment. Access to specialist gynaecology, andrology and fertility follow‐up care with psychological support as part of long‐term survivorship care is inconsistent, with patients at risk of loss to follow‐up particularly when transitioning from paediatric to adult care teams.

Fertility preservation for medical indications should be considered an essential aspect of care for individuals at risk of treatment induced fertility. As childhood cancer survival rates improve across the globe, fertility preservation is an increasingly important issue. Despite growing awareness, access remains inequitable. Without national coordination, an individual's demographics may determine if they can access fertility preservation or not. Establishing nationally commissioned services would improve access and transparency. These services should provide clear, timely referral pathways, ensure sustainable funding, and be supported by a coordinated network with robust data collection and clinical governance. Without careful design, disparities in accessing services will remain and young people may miss the opportunity to preserve their fertility.

## Author Contributions

The UK Young People's Fertility Consortium represents a multidisciplinary group of specialists from across the United Kingdom with expertise in fertility preservation for young people. Christian M. Becker and Sheila Lane conceived the idea for the manuscript. Ursula Blyth drafted and wrote the initial manuscript with support from Pedro Melo. All authors contributed to sections of text, reviewed and edited multiple versions, and approved the final version.

## Funding

The authors have nothing to report.

## Conflicts of Interest

The authors declare no conflicts of interest.

## Data Availability

Data sharing not applicable to this article as no datasets were generated or analysed during the current study.
